# Pilot study of atomoxetine in patients with Parkinson’s disease and dopa-unresponsive Freezing of Gait

**DOI:** 10.1186/s40035-015-0047-8

**Published:** 2015-12-10

**Authors:** Gonzalo J. Revuelta, Aaron Embry, Jordan J. Elm, Chris Gregory, Amy Delambo, Steve Kautz, Vanessa K. Hinson

**Affiliations:** Movement Disorders Division, Department of Neurology, College of Medicine, Medical University of South Carolina, 208B Rutledge Avenue, MSC 108, Charleston, SC 29425 USA; Department of Health Sciences and Research, Center for Rehabilitation Research in Neurological Conditions, College of Health Professions, Medical University of South Carolina, Charleston, SC USA; Department of Public Health Sciences, College of Medicine, Medical University of South Carolina, Charleston, SC 29425 USA; Ralph H. Johnson VA Medical Center, Charleston, SC USA

**Keywords:** Parkinson’s disease, Freezing of gait, Noradrenaline, Atomoxetine, Dopa-response

## Abstract

**Background:**

Freezing of gait (FoG) is a common and debilitating condition in Parkinson’s disease (PD) associated with executive dysfunction. A subtype of FoG does not respond to dopaminergic therapy and may be related to noradrenergic deficiency. This pilot study explores the effects of atomoxetine on gait in PD patients with dopa-unresponsive FoG using a novel paradigm for objective gait assessment.

**Findings:**

Ten patients with PD and dopa-unresponsive FoG were enrolled in this eight-week open label pilot study. Assessments included an exploratory gait analysis protocol that quantified spatiotemporal parameters during straight-away walking and turning, while performing a dual task. Clinical, and subjective assessments of gait, quality of life, and safety were also administered. The primary outcome was a validated subjective assessment for FoG (FOG-Q). Atomoxetine was well tolerated, however, no significant change was observed in the primary outcome. The gait analysis protocol correlated well with clinical scales, but not with subjective assessments. DBS patients were more likely to increase gait velocity (*p* = 0.033), and improved in other clinical assessments.

**Conclusions:**

Objective gait analysis protocols assessing gait while dual tasking are feasible and useful for this patient population, and may be superior correlates of FoG severity than subjective measures. These findings can inform future trials in this population.

## Findings

### Background

Freezing of gait (FoG) is a common and disabling symptom for patients with Parkinson’s disease (PD). FoG may respond to dopaminergic therapies and DBS early in the course, of PD, and later become dopa-unresponsive [1, 2]. Noradrenergic deficiency has been well documented in PD and has long been proposed as a potential etiology for FoG [3], in addition attention deficit and executive dysfunction have also been strongly associated with FoG [4, 5]. However, multiple trials of noradrenergic medications have yielded conflicting results [3]. There is currently no accepted objective measure of FoG severity.

Atomoxetine (ATM) is a norepinephrine reuptake inhibitor shown to improve attention deficit in adults [6] and executive dysfunction in PD [7], with reports of improvements in FoG [8]. In this study we explore the effects of ATM on multiple gait parameters in patients with PD who experience dopa-unresponsive FoG using multiple assessments as potential outcome measures of FoG. The purpose of this study is to gather pilot data to be used to aid in the design of larger randomized clinical trials of therapeutic agents for the treatment of dopa-unresponsive FoG.

### Methods

#### Subjects

Subjects (ages 18–80) with PD (Hoehn and Yahr stage 2–4) and dopa-unresponsive-FoG were recruited for this study. All patients met UK-Brain Bank criteria for idiopathic PD, had a positive response to item 14 of the Unified Parkinson’s Disease Rating Scale (UPDRS), and were observed to have actual FoG at screening, in the on state. Subjects must be on stable medications for 3 months prior to starting the study, and had to be able to walk 20 feet without an assistive device. Subjects who were intolerant or hypersensitive to the drug class, were on monoamine oxidase inhibitors, were demented (MMSE < 26), whose gait dysfunction was attributable to other conditions, had narrow angle glaucoma, pheochromocytoma, severe cardiovascular conditions, uncontrolled hypertension, symptomatic tachyarrhythmias, uncontrolled depression or suicidal ideation, were excluded from the study. All subjects underwent medical clearance prior to enrolling in the study. The Institutional Review Board of the Medical University of South Carolina approved the study and investigational new drug (IND) exception was granted by the Food and Drug Administration.

#### Study design

This was an open-label, forced titration, 8-week study to explore the safety, tolerability and efficacy of atomoxetine for the treatment of dopa-unresponsive FoG in patients with PD. A 3-point change in the Freezing of Gait Questionnaire (FOG-Q) was chosen as the primary efficacy outcome measure in order to power this pilot study. A sample size of 10 patients treated with atomoxetine would have 80 % power to detect a pre-post reduction in the mean FOG-Q score of 2.6, assuming a standard deviation of the difference of 3.0, using a paired *t*-test with a 0.05 one-sided significance level. The estimate of the standard deviation of the change was based on literature [9]. Exploratory efficacy outcome measures were: changes in spatiotemporal parameters while performing a dual cognitive task, reduction in falls, clinical global improvement (CGI) and changes in clinical gait outcome measures (see gait assessments). Fisher’s exact test was used to test the null hypothesis that the proportion of responders was the same for patients who received DBS or did not.

All patients underwent a screening visit prior to enrollment. Patients who enrolled in the study were started on 40 mg daily for two weeks then increased to 40 mg twice daily for the 4-week treatment period. They were then reduced to 40 mg daily for one week, and washed out for two weeks. Full gait evaluations occurred at baseline (Visit 2), after the 4-week treatment period at full dose (Visit 4), and after washout (Visit 5). Visit 3 was a safety only visit without gait assessments.

### Safety and tolerability

Safety was assessed by reported adverse events, UPDRS parts 1–4 scores, Falls Efficacy (FES) questionnaire, EKG, vital sign assessments, and liver function tests.

#### Gait assessments

Gait assessments were performed in the “relative on state” defined as 1–2 h after taking their medication, with their DBS on (when applicable), and patient report of feeling in the on state. The FOG-Q was administered with each gait assessment at baseline (Visit 2), after the 4-week treatment period at full dose (Visit 4), and after washout (Visit 5).

##### Clinical evaluations

The Dynamic Gait Index (DGI) and the Tinetti Gait and Balance assessments were administered. Falls incidence and FES were also administered.

##### Spatiotemporal parameters

**Fig. 1:**
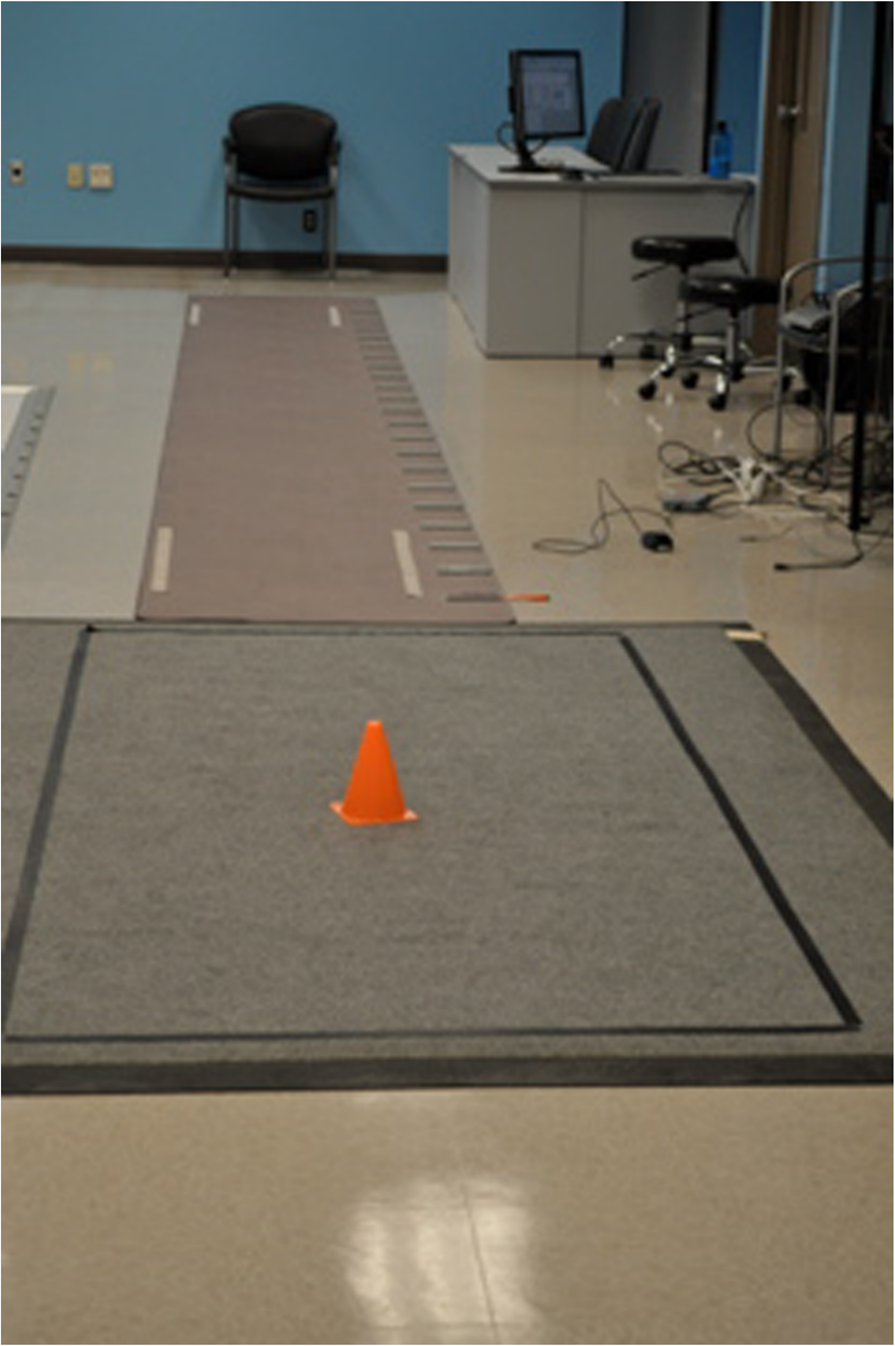
Electronic Walkway Setup. The GaitRite electronic walkway is shown here, connected to the M2 electronic walkway demonstrating the path patients took during study procedures to collect spatiotemporal parameters, including the turn task

All patients received brief training regarding the protocol prior to initial assessment. Subjects were asked to walk on an electronic walkway composed of the GaitRite attached to the M^2^ (See Fig. 1). Each subject was instructed to stand from a chair, walk the length of the GaitRite walkway, turn 180° on the M^2^ and return to the seated position at the end of the GaitRite. Patients were then asked to repeat this task while also performing a concurrent cognitive task. One of two cognitive tasks was used (serial 7’s, or alternating letters of the alphabet). Care was taken to start at a different number or letter so as to reduce any practice effect on the difficulty of the cognitive task. Performance on the cognitive tasks were rated as previously described [10].

### Results

Ten patients who met all inclusion and exclusion criteria were enrolled in the study. There were no early withdrawals or terminations from the study. The mean age was 67.1 years, there were eight males, and three patients were status post bilateral subthalamic nucleus DBS.

#### Safety and tolerability

All ten patients tolerated the study drug well; there were no drug discontinuation, drug dose adjustments, or withdrawals. Seven of ten patients had eight adverse events, all of which were mild. Three patients had mild increases in creatine which subsided on repeat testing. Miscellaneous reports of increase in creatine (3), worsening FoG (1), diarrhea (1), worsening dyskinesia (1), nausea (1), were recorded but resolved spontaneously, are were not deemed clinically significant or related to study drug. Another subject reported feeling mildly “jittery” and improved after washout. This was not deemed clinically significant but was likely related to study drug. There were no observed increases in blood pressure, EKG changes, palpitations, arrhythmias, or changes in liver function. There was no change in UPDRS scores to suggest worsening of motor symptoms.

#### Gait assessments

There was no significant change in the primary outcome measure (FOG-Q) between baseline and treatment visits in this cohort as a whole (*p* = 0.97). There were also no significant changes in clinical assessments, falls incidence, or CGI scores, before and after treatment (See Table 1). As a group there was no significant change in spatiotemporal parameters with or without concurrent dual task performance before and after treatment (see Table 2).

**Table 1:** Changes in clinical scales pre and post treatment

	Baseline		Post treatment		Post washout	
	Mean	SD	Mean	SD	Mean	SD
FOGQ	12.50	2.95	12.10	3.11	13.10	2.64
CGI	3.60	1.26	3.00	1.49	4.00	1.33
DGI	19.70	2.63	19.60	4.35	19.50	5.38
Tinetti	23.10	4.65	23.90	4.31	25.00	3.33
FES	31.40	19.74	23.60	16.06	24.00	13.05
PDQ39	26.40	17.41	22.31	12.83	n/a	n/a
UPDRS I	2.50	3.06	2.10	2.56	1.78	2.05
UPDRS II	16.10	5.26	15.40	5.58	15.56	6.67
UPDRS III	22.80	9.95	21.70	10.57	23.90	9.78
UPDRS total	41.40	14.66	39.20	15.65	42.22	15.63

Data correspond to scores on clinical scales for gait, balance, freezing of gait, falls, Parkinson’s symptoms, and quality of life. For all scales lower is better except for the Tinnetti

*FOG-Q* Freezing of Gait Questionnaire, *CGI* Clinical Global Impression, *DGI* Dynamic Gait Index, Tinnetti Gait and Balance, *FES* Falls Efficacy Scale, *PDQ-39* Parkinson’s Disease Questionnaire (quality of life measure), *UPDRS* (Unified Parkinson’s Disease Rating Scale), Part I (Mentation, Behavior, Mood), Part II (Activities of Daily Living), Part III (Motor), Part IV (Complications from therapy)

**Table 2:** Changes in spatiotemporal parameters pre and post treatment

	Baseline		Post treatment		Post washout	
	Mean	SD	Mean	SD	Mean	SD
Velocity	91.30	19.81	96.69	25.24	94.79	28.21
Cadence	116.4	11.56	120.78	11.09	117.24	10.99
Step length	47.62	11.55	48.77	13.51	48.42	12.85
Stride length	95.56	23.17	97.74	27.00	97.30	25.71
SST	67.43	2.24	67.49	3.16	68.65	3.83
DST	32.57	2.28	32.52	3.15	31.36	3.83

Data correspond to objective spatiotemporal parameters collected during the “timed up and go” task, including “time to turn” as collected by the two consecutive electronic walkways described on Fig. 1, at baseline visit (prior to treatment with atomoxetine), Visit 4 (post treatment) and Visit 5 (post washout)

*SD* standard deviation, *SST* single support time, *DST* double support time

FOG-Q scores did not correlate with values for objective spatiotemporal parameters with or without concurrent dual task performance, including time to turn. However, time to turn was highly correlated with clinical measures of gait severity including: Dynamic Gait Index (*p* = 0.006) and Tinetti (*p* = 0.012); as well as with PD severity scales including: UPDRS motor (*p* = 0.02) and total score (*p* = 0.056). In most instances dual tasking improved the association further: Dynamic Gait Index (*p* = 0.001), UPDRS motor (*p* = 0.013) and total score (*p* = 0.028).

We controlled for performance on cognitive abilities by rating their ability to perform the cognitive tasks while walking. There was no consistent improvement or worsening in their performance in these tasks ensuring there was no significant practice effect and there was a consistent difficulty level across visits.

A post-hoc analysis was performed where a subgroup who had a greater than 0.09 m/s improvement in velocity from baseline to the post treatment visit in their self-selected walking speed was further evaluated. This cutoff was based on published minimal detectable change for self-selected walking speed for PD patients [11]. Four patients were found to have increased walking speed based on these criteria. All three DBS “responded” and in fact, DBS patients were more likely to have improvement beyond 12 cm/s in velocity (*p* = 0.033). Further analysis of this subgroup revealed a decrease in FOG-Q scores greater than 3 points (pre-specified clinically significant change) as well as significant decreases in CGI score (2 points) and PDQ-39 scores (12.61 points). This group of patients also had a reduction in time to turn with and without a concurrent cognitive task.

### Conclusions

We report the results of an eight-week open label study designed to explore the effects of atomoxetine on gait parameters in patients with PD and dopa-unresponsive FoG. Since dopa-unresponsive FoG is by definition not a dopaminergic phenomenon, a noradrenergic agent was chosen, and gait assessments were designed to capture attention deficit and executive dysfunction by utilizing increased cognitive load paradigms. The study did not meet its primary endpoint of an improvement of three points on the FOG-Q, however, important observations were made. Atomoxetine was safe and well tolerated in this patient population. DBS patients were more likely to have clinically significant increases in walking speed, FoG (as assessed by FOG-Q), time to turn while dual tasking, and quality of life (as assessed by PDQ-39). This may be explained by the fact that DBS patients who have good control of other PD motor symptoms (dopa-responsive symptoms) and still freeze provide a particularly good model for the target population of this study (dopa-unresponsive freezers).

Regarding the spatiotemporal data we report the feasibility of this protocol for the quantification of gait parameters, including time to turn, on two electronic walkways. Furthermore, we found time to turn to be a strong correlate of gait disturbance severity in this cohort, particularly under the dual task condition, and found no correlation between any of the measured objective parameters and the FOGQ. This may be related to the subjective nature of this measure.
